# Polarization-dependent interfacial coupling modulation of ferroelectric photovoltaic effect in PZT-ZnO heterostructures

**DOI:** 10.1038/srep22948

**Published:** 2016-03-08

**Authors:** Dan-Feng Pan, Gui-Feng Bi, Guang-Yi Chen, Hao Zhang, Jun-Ming Liu, Guang-Hou Wang, Jian-Guo Wan

**Affiliations:** 1 National Laboratory of Solid State Microstructures and Department of Physics, Nanjing University, Nanjing 210093, China; 2 Collaborative Innovation Center of Advanced Microstructures, Nanjing University, Nanjing 210093, China; 3 Department of Physics and Astronomy, University of Kentucky, Lexington, Kentucky 40506-0055, USA

## Abstract

Recently, ferroelectric perovskite oxides have drawn much attention due to potential applications in the field of solar energy conversion. However, the power conversion efficiency of ferroelectric photovoltaic effect currently reported is far below the expectable value. One of the crucial problems lies in the two back-to-back Schottky barriers, which are formed at the ferroelectric-electrode interfaces and blocking most of photo-generated carriers to reach the outside circuit. Herein, we develop a new approach to enhance the ferroelectric photovoltaic effect by introducing the polarization-dependent interfacial coupling effect. Through inserting a semiconductor ZnO layer with spontaneous polarization into the ferroelectric ITO/PZT/Au film, a p-n junction with strong polarization-dependent interfacial coupling effect is formed. The power conversion efficiency of the heterostructure is improved by nearly two orders of magnitude and the polarization modulation ratio is increased about four times. It is demonstrated that the polarization-dependent interfacial coupling effect can give rise to a great change in band structure of the heterostructure, not only producing an aligned internal electric field but also tuning both depletion layer width and potential barrier height at PZT-ZnO interface. This work provides an efficient way in developing highly efficient ferroelectric-based solar cells and novel optoelectronic memory devices.

It is well known that ferroelectric materials have important applications in many fields thanks to their switchable polarization response to external electric fields^1^,^2^. The recent six years have witnessed another potential value of ferroelectrics in the photovoltaic area^3^,^4^,^5^,^6^,^7^. Ferroelectric photovoltaic effect (FePVE) has drawn much attention in the field of solar-energy conversion because of large above-bandgap photo-voltages. For instance, a 15 V open circuit voltage can be obtained in BiFeO_3_ thin films, much greater than the 1.1 V limit of the Si p-n junction^8^,^9^. In a typical electrode/ferroelectric/electrode structure, the separation of photo-induced carriers is mainly dominated by two factors, i.e. the ferroelectric polarization-induced internal electric field (i.e. the depolarization field) and two back-to-back build-in electric fields of Schottky barriers formed at the ferroelectric-electrode interfaces^10^. Since most ferroelectrics have relatively large bandgaps (e.g. over 3.5 eV for Pb(Zr,Ti)O_3_, PZT)^11^ and the two interfacial build-in electric fields usually have the opposite directions in PZT-based heterostructures, the power conversion efficiency (PCE) is too low to meet practical applications. In order to overcome these problems, many efforts have been made in the past few years. Designing heterostructures composed of ferroelectrics and narrow bandgap semiconductors (e.g. Ag_2_O^12^, Cu_2_O^13^ and amorphous Si^14^^ ^etc.) has been demonstrated to be a low-cost and feasible way of enhancing the PCE of FePVE, which should meet the following principles. The energy band of the semiconductor layer must match well with the ferroelectric layer so that the direction of the interfacial electric field at the ferroelectric-semiconductor interface is the same as that of the build-in electric field at ferroelectric-electrode interface, and the semiconductor-electrode interface should be ohmic or quasi-ohmic contact.

Therefore, it has occurred to us that if the semiconductor layer is also polarized, an extra internal field induced by this polar semiconductor might improve the PCE of the heterostructure^15^. As a typical n-type semiconductor with hexagonal wurtzite structure, ZnO not only exhibits piezoelectric (stress- or strain-induced) effect but also has a spontaneous polarization of about 4.1–7.0 μC/cm^2^ 16. Since the spontaneous polarization of ZnO originates from the asymmetric permanent static electric dipole distribution within the unit cell, it cannot be altered by an external electric field^17^. In previous investigations, the polarization-dependent interfacial effects have been utilized to control the resistive switching behaviors in some ferroelectric/semiconductor heterostructures (e.g. BaTiO_3_/ZnO^18^, BiFeO_3_/ZnO^19^, PZT/ZnO^20^, etc.) by tuning accumulated or depleted charge carriers at the interface through external bipolar electric fields. However, few investigations have reported on the influence of such interfacial coupling on the photovoltaic effect. Since the polarization-dependent interfacial coupling can give rise to a change in band structure of the ferroelectric-semiconductor heterostructure, an improvement of FePVE might be expected.

In this work, we introduce the polarization-dependent interfacial coupling effect to the ferroelectric PZT film by inserting a semiconductor ZnO layer. A great enhancement of FePVE is observed in such heterostructure and a controllable FePVE modulation is realized through changing the interfacial band structures by poling voltages. We demonstrate that after inserting a ZnO layer into ITO/PZT/Au, the PCE of the heterostructure is nearly two orders of magnitude higher than the initial one. Moreover, the polarization modulation ratio of PCE in the heterostructure is enhanced about four times. Based on systematic measurements of photovoltaic current-voltage curves at various polarization states and detailed analysis of energy band diagrams, we not only confirm the existence of polarization-dependent interfacial coupling in the heterostructure, but also discover how ZnO polarization influences the FePVE of the heterostructure.

## Results and Discussion

**Figure 1:**
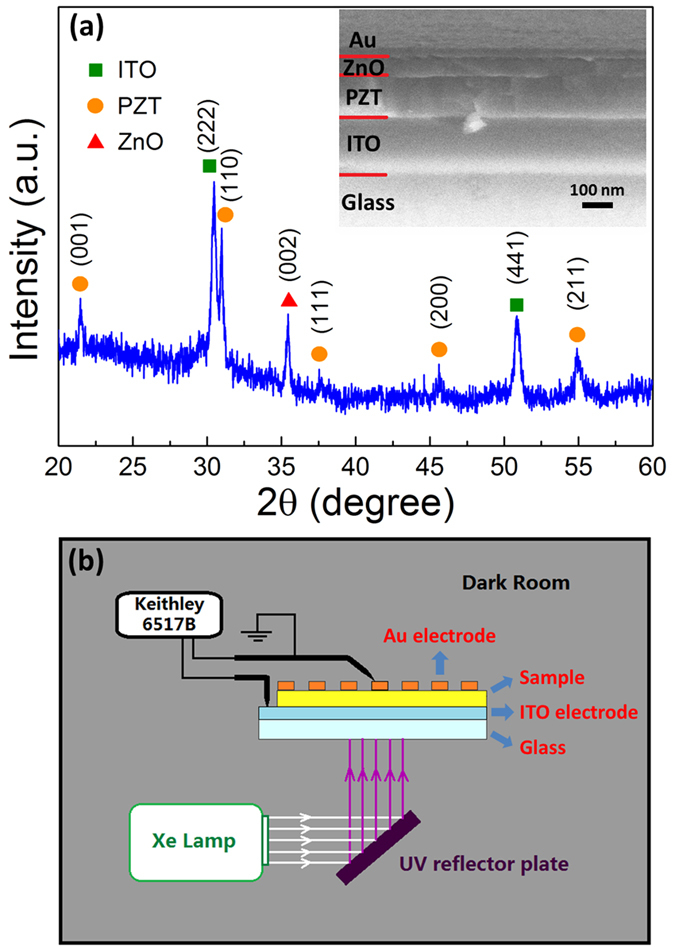
(**a**) XRD pattern of the ITO/PZT/ZnO/Au heterostructure. The inset shows the cross-sectional SEM image. (**b**) A sketch map of the ferroelectric photovoltaic measurement apparatus.

The heterostructure was prepared using a sol-gel process by depositing PZT and ZnO films in sequence on the ITO-coated glass substrate (see Experimental Section for details). For electrical measurements, a 40 nm Au layer was deposited onto the heterostructure as top electrode by an ion sputtering process. Figure 1(a) shows the X-ray diffraction data for a typical ITO/PZT/ZnO/Au heterostructure. The polycrystalline PZT perovskite phase and preferred (002)-oriented ZnO wurtzite phase are observed. No impurity phase is found except for these two phases. The preferred orientation in ZnO layer indicates the existence of static electric dipoles along the *c* axis of ZnO lattice, which causes the formation of intrinsic spontaneous polarization^17^. The inset of Fig. 1(a) presents the cross-sectional SEM image of the sample. The interfaces between each layer are distinguishable, and the thicknesses of the PZT and ZnO layers are about 130 nm and 60 nm, respectively. For comparison, ITO/PZT/Au and ITO/ZnO/PZT/Au heterostructures were also prepared, in which the thicknesses of the PZT and ZnO layers were the same as that of ITO/PZT/ZnO/Au.

**Figure 2:**
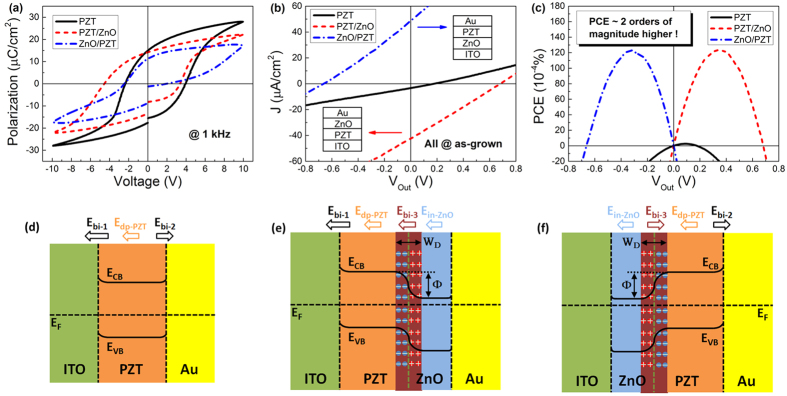
(**a**) Polarization-voltage hysteresis loops of the samples measured at 1 kHz. (**b**) Comparison of photovoltaic current density (J) vs output voltage (V_out_) curves of the as-grown samples. (**c**) Calculated power conversion efficiency (PCE) as a function of V_out_. (**d**–**f**) Schematic energy band diagrams and internal electric fields distribution of three as-grown samples, i.e. ITO/PZT/Au, ITO/PZT/ZnO/Au and ITO/ZnO/PZT/Au. Note that the two electrodes of three samples are all short-circuited.

The heterostructure in which the ZnO layer was inserted between PZT layer and Au electrode (i.e. ITO/PZT/ZnO/Au) was first studied. In the as-grown sample, there exist both the intrinsic spontaneous polarization in ZnO layer and the initial ferroelectric polarization in PZT layer caused by self-polarization effect^10^, of which the latter polarization can be altered by external poling voltages while the former cannot. We measured polarization vs. voltage hysteresis loops for the sample, as depicted in Fig. 2(a). The evident asymmetry of coercive electric field (E_c_) is observed in ITO/PZT/Au, which should be attributed to the difference of work functions between ITO and Au electrodes as well as the migration of defect charges caused by the high temperature annealing process of bottom ITO electrodes^21^,^22^. This asymmetry of E_c_ has a big shift towards the opposite direction when the ZnO layer is inserted. Since E_c_ reflects the ability of bonded polarization charges to inverse with external electric fields^1^, this shift is actually a signature of the occurrence of polarization-dependent interfacial coupling effect. Besides, the positive E_c_ shifted closer to zero reflects a predictable band shift due to the ZnO’s polarization.

It is assumed that the interfacial coupling effect may play an important role in modulating the FePVE of the PZT-ZnO heterostructure through varying the energy band structure. So we subsequently measured the photovoltaic effect for the as-grown samples. For accurate measurements, the whole apparatus was placed in a dark room, as shown in Fig. 1(b). Considering that the bandgaps of both PZT and ZnO are above 3.0 eV, we used UV light to irradiate the samples so that sufficient photo-induced carriers can be generated. The spectra of the incident UV light is shown in Figure S1 of Supporting Information (SI). The intensity of the incident light was 60 mW/cm^2^. Figure 2(b) presents photovoltaic current density vs output voltage (J-V_out_) curves for the as-grown samples. Compared with the ITO/PZT/Au, the as-grown ITO/PZT/ZnO/Au exhibits significant improvements in both short circuit current density (J_sc_) and open circuit voltage (V_oc_). As shown in the red dashed curve, V_oc_ increases from 0.178 to 0.681 V while J_sc_ rises from −3.345 to −42.409 μA/cm^2^. Figure 2(c) further gives corresponding PCE vs output voltage (PCE-V_out_) curves. The maximum PCE value of the ITO/PZT/ZnO/Au heterostructure appears at V_out_ = +0.35 V, about 90 times of that in ITO/PZT/Au.

The great improvement of PCE in the ITO/PZT/ZnO/Au heterostructure can be understood based on the analysis of simplified energy band diagrams, as shown in Fig. 2(d,e). To draw the energy band diagrams, we did a series of complementary experiments so as to estimate the depolarization field (E_dp_) and internal field directions of the as-grown PZT and ZnO layers, to determine the optical bandgaps and the contact formation at different interfaces (see Figures S2–S4 for details, SI). The details on the determination of the energy band diagram for the PZT/ZnO bilayer is presented in Section V of SI^20^. In nanoscale ferroelectrics, the depolarization field arises at an interface, stemming from the incomplete screening of the polarization charges. The direction of the depolarization field is always against the polarization and changes sign by reversing the polarization. For the as-grown ITO/PZT/Au (Fig. 2(d)), the weak polarization is directed to the top Au electrode (Section II, SI), so the direction of the depolarization field points to the bottom ITO electrode. The initial polarization in PZT layer produces a small depolarization field (E_dp-PZT_)^23^, which is helpful for separating photo-induced carriers. However, the Schottky barriers at the ITO/PZT and PZT/Au interfaces induce two back-to-back build-in electric fields E_bi–1_ and E_bi–2_, one of which shares the opposite direction of the E_dp-PZT_ and hinders the transportation of photo-induced carriers to the outside circuit^13^. So the PCE value is quite low. This situation is greatly improved in ITO/PZT/ZnO/Au where the ZnO layer plays a crucial role in tuning the band structure of the heterostructure. As shown in Fig. 2(e), due to good matching of energy bands between ZnO and Au, the ZnO/Au interface exhibits (quasi) ohmic contact (Figure S4(c)). So one of the two back-to-back build-in electric fields (i.e. E_bi-2_) disappears. Meanwhile, the spontaneous polarization in ZnO layer causes an additional internal field (E_in-ZnO_) with the same direction of E_dp-PZT_, further boosting the ability of separating photo-induced carriers. Moreover, the contact between p-type PZT and n-type ZnO layers brings about a p-n junction at the interface, producing an extra build-in electric field (E_bi-3_) with the same direction of E_bi-1_. Most importantly, since ZnO and PZT layers own spontaneous and remnant polarizations respectively, the polarization-dependent interfacial coupling effect at the PZT-ZnO interface occurs, leading to a change in energy band structure. To be specific, the interfacial polarization bound charges attract free carriers in both PZT and ZnO layers to be accumulated or depleted at the PZT-ZnO interface, inducing the bending of interfacial energy band^24^. It is expected that the variation of such interfacial coupling strength will give rise to a change in FePVE of the heterostructure.

**Figure 3:**
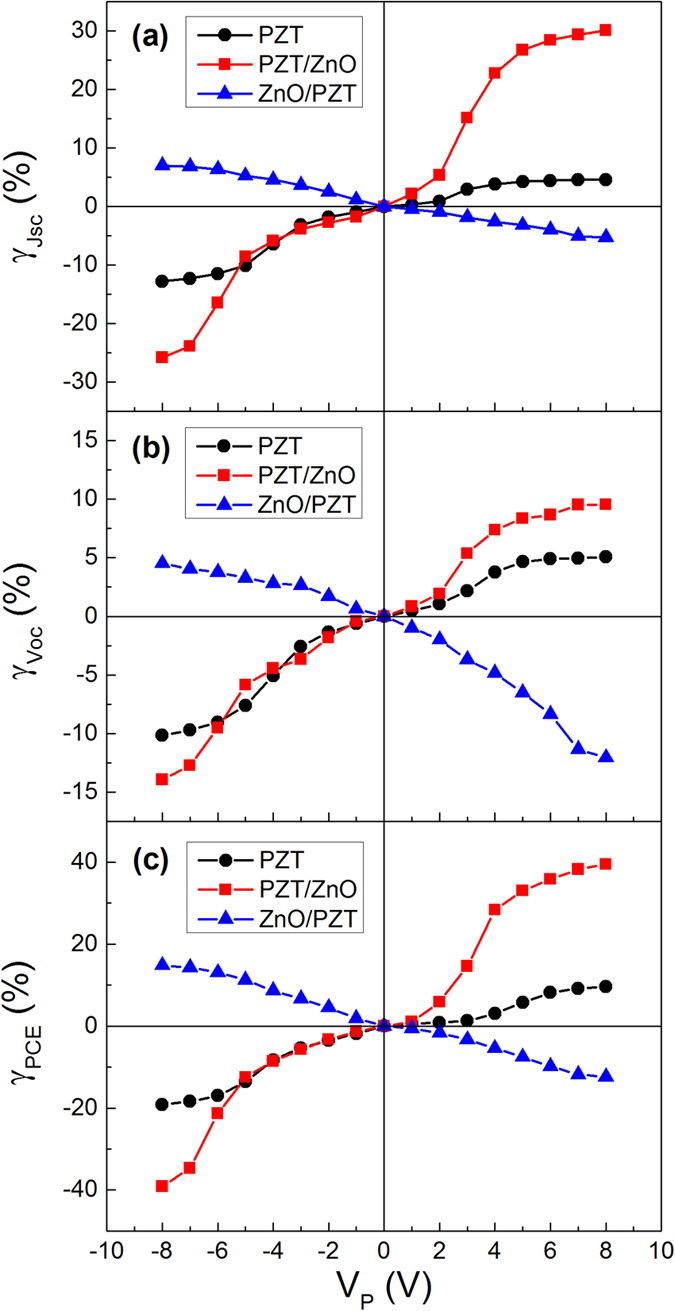
The polarization modulation ratio (γ) of (**a**) J_sc_, (**b**) V_oc_, and (**c**) PCE as a function of poling voltage (V_P_). Note that each point represents the value summarized from the corresponding photovoltaic J-V_out_ curves after the samples are poled at a given V_P_ value.

Accordingly, we further studied the influence of polarization-dependent interfacial coupling strength on the FePVE of ITO/PZT/ZnO/Au. To vary the coupling strength between PZT and ZnO layers, the as-grown heterostructures were poled at various voltages. After that, the photovoltaic J-V_out_ curves at different polarization states were measured. The corresponding J_sc_, V_oc_ and PCE values were also calculated. As is known to all, the photovoltaic process consists of three parts: photo-generation, separation of photo-induced carriers and the current collection. The change in every single process can lead to the variation of PCE. Considering that the photo-generation part is mainly related to the light absorption of the heterostructure, and when the preparation of the heterostructure is done the photo-generation part of FePVE cannot be changed, so the FePVE is mostly ascribed to the charge separation process, which changes with the variation of the polarization state (dependent on the direction and magnitude) in the PZT layer. Here we define the modulation ratio for these parameters at a given polarization state as:


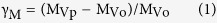


where M_Vp_ represents the J_sc_, V_oc_ or PCE value after the sample is subjected to the poling voltage V_P_ , M_Vo_ is the corresponding value at V_P_ = 0 (i.e. at as-grown state). Figure 3 presents the modulation ratio of J_sc_, V_oc_ and PCE as a function of V_P_ for the samples. In ITO/PZT/Au, no interfacial coupling exists, so the modulation of FePVE is dominated by the ferroelectric polarization in PZT layer. All the J_sc_, V_oc_ and PCE values only increase slightly with increasing the poling voltage. Typically, at V_P_ = +8 V (the PZT layer is at saturation polarization state), the modulation ratios of J_sc_, V_oc_ and PCE only reach 5%, 4%, and 9%, respectively. Comparatively, after inserting a ZnO layer between PZT layer and Au electrode, the modulation ratios of J_sc_, V_oc_ and PCE have a large change, e.g. the modulation ratios of J_sc_, V_oc_ and PCE at V_P_ = +8 V reach as high as 30%, 9% and 40% respectively, all of which are several times larger than those of ITO/PZT/Au. On the contrary, at negative polarization states, the modulation ratios of J_sc_, V_oc_ and PCE decrease dramatically. For instance, the modulation ratios of J_sc_, V_oc_ and PCE at V_P_ = −8 V drops to −25%, −15% and −40%, respectively. The above results distinctly imply that the polarization-dependent interfacial coupling effect has great influence on the FePVE of the heterostructure.

**Figure 4:**
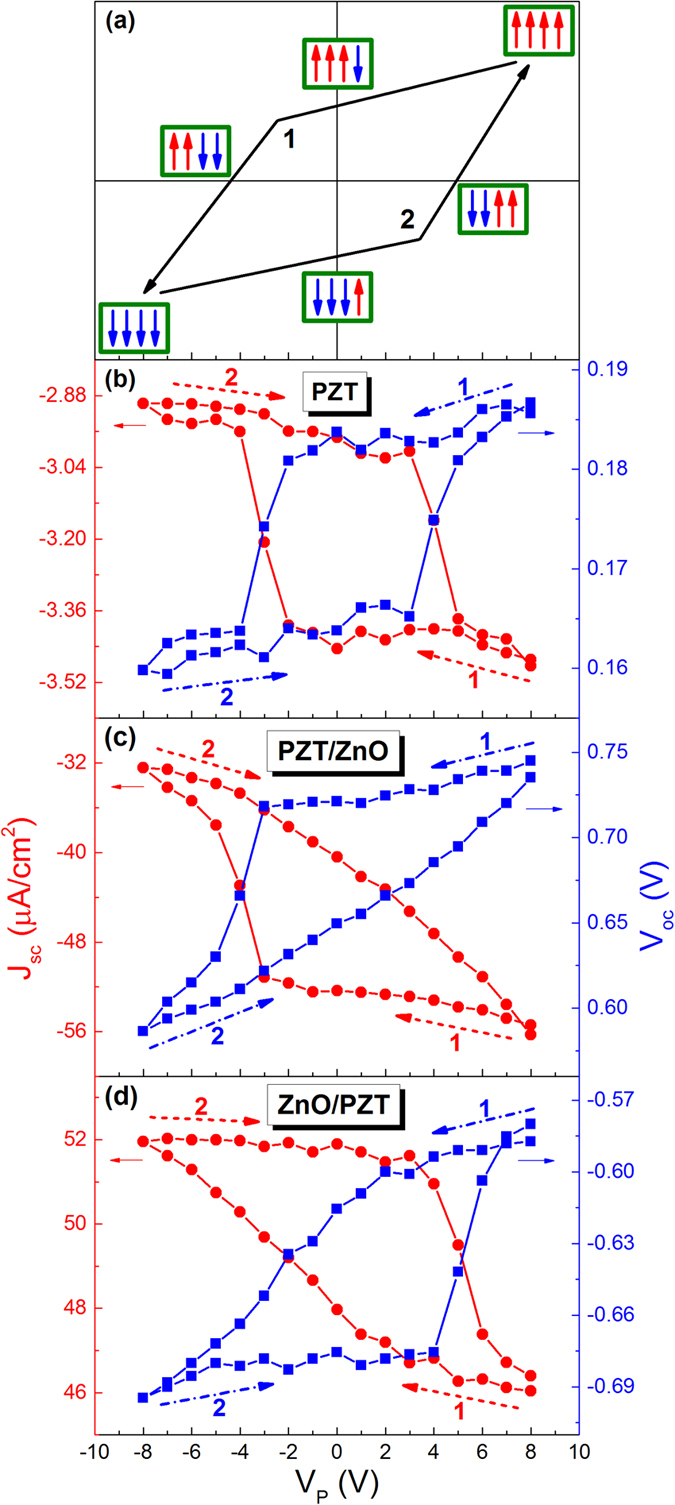
(**a**) The sequence of applying external poling voltage: +8 V → 0 → −8 V → 0 → +8 V, which is marked by the arrow polylines 1 and 2. The up and down arrows in green rectangles represent the schematic ferroelectric domain directions in the PZT layer. Note that the direction of spontaneous polarization in ZnO layer is not changed by external applied voltages. (**b**–**d**) The photovoltaic V_oc_ and J_sc_ hysteresis loops of three samples as a function as poling voltage (V_P_): (**b**) ITO/PZT/Au, (**c**) ITO/PZT/ZnO/Au and (**d**) ITO/ZnO/PZT/Au.

The dependence of photovoltaic output on the polarization-dependent interfacial coupling effect was further explored by measuring photovoltaic J_sc_-V_p_ and V_oc_-V_p_ hysteresis loops of the heterostructure. Figure 4(a) schematically gives the sequence of applying the poling voltage V_p_, i.e. +8 V → 0 → −8 V → 0 → +8 V. By measuring photovoltaic J-V_out_ curves at each polarization state, the corresponding J_sc_ and V_oc_ values were obtained. For the ITO/PZT/Au shown in Fig. 4(b), both J_sc_-V_p_ and V_oc_-V_p_ loops exhibit symmetric hysteresis characteristics similar to a typical ferroelectric loop, indicating that the FePVE of ITO/PZT/Au is closely related to the ferroelectric polarization in PZT layer^25^. Conversely, after inserting a ZnO layer between PZT layer and Au electrode, both V_oc_-V_p_ and J_sc_-V_p_ loops become much asymmetric (Fig. 4(c)). At the second half stage of −8 V → 0 → +8 V, the hysteresis characteristic of V_oc_ (or J_sc_) -V_p_ loop disappears completely, which is replaced by an almost linear dependence relationship. It is another solid evidence of the polarization-dependent interfacial coupling effect between PZT and ZnO layers.

**Figure 5:**
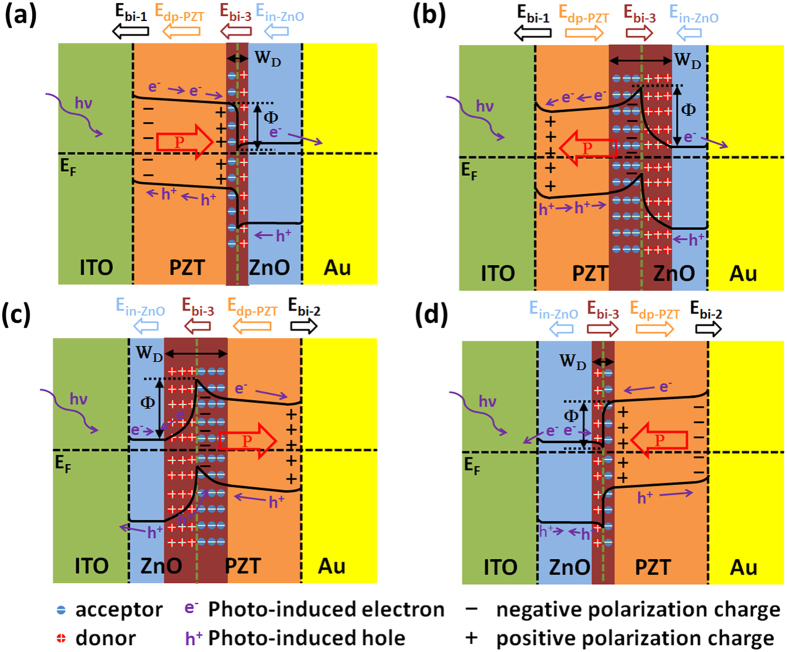
The whole energy band diagrams of two kinds of heterostructures after poled by +8 V or −8 V when the heterostructures are under illumination and all the two electrodes are short-circuited. (**a**,**b**) The ITO/PZT/ZnO/Au heterostructure poled by (**a**) +8 V and (**b**) −8 V; (**c**,**d**) The ITO/ZnO/PZT/Au heterostructure poled by (**c**) +8 V and (**d**) −8 V. In each energy band diagram, the internal electric fields distribution and the generation, separation and motion of photo-induced carriers under incident light are diagrammatically presented.

To understand such unusual photovoltaic phenomenon, we compared the energy band diagrams of the ITO/PZT/ZnO/Au heterostructure at three typical polarization states, as shown in Figs 5(a,b) and 2(e). According to previous studies^26^,^27^, the polarization can effectively induce band bending at the interfaces between ferroelectrics and metal (or oxide). When the ITO/PZT/ZnO/Au heterostructure is at positive polarization state (defined as P+, as shown in Fig. 5(a)), the E_dp-PZT_ in PZT layer becomes larger with increasing the poling voltage in comparison with the as-grown state. Meanwhile, more positive polarization charges aggregate at the PZT-ZnO interface^28^, attracting abundant of negative majority free carriers in ZnO layer and driving more space charges (e.g. defect charges and oxygen vacancies) to the surface of PZT layer^29^. As a result, the depletion layer width (W_D_) of the p-n junction decreases while the E_bi-3_ increases^30^,^31^. In contrast, if the sample is at the negative polarization state (defined as P-, as shown in Fig. 5(b)), the negative majority free carriers in ZnO layer and the space charges in PZT layer are repelled by the negative polarization charges at the interface, which leads to an increase of W_D_. At the same time, an energy band bending is induced in the depletion layer because of the incomplete screening of the bound polarization charges, leading to a great increase of potential barrier height (Ф) at PZT-ZnO interface^32^. Based on the above model, it is easily understood that the photovoltaic J_sc_-V_p_ and V_oc_-V_p_ hysteresis loops in ITO/PZT/ZnO/Au are different from those in ITO/PZT/Au. During the first half poling stage (+8 V → 0 → −8 V, Fig. 4(c)), when the electric field is close to the negative coercive field, partial upward polarization states start to reverse to the downward, causing the formation of peak potential barrier at the PZT-ZnO interface (Fig. 5(b)). So W_D_ becomes wider due to the high mobility of free electrons in ZnO layer^16^. Therefore, both J_sc_ and V_oc_ values drop sharply. While during the second half poling stage (−8 V → 0 → +8 V, Fig. 4(c)), due to the polarization-dependent interfacial coupling between ZnO and PZT layers, more and more negative majority carriers accumulate at the interface when external poling voltage increases. As a result, both Ф and W_D_ values decreases bit by bit, causing both J_sc_ and V_oc_ vary almost linearly with increasing the poling voltage. In addition, it is worth noting that the polarization-dependent interfacial coupling between ZnO and PZT layers not only depends on the magnitude of the polarization in the PZT layer, but also is related to the direction of the polarization in the PZT layer, i.e. its influence on the FePVE becomes different when the polarization direction of PZT layer is opposite. For instance, when the polarization direction of PZT layer points to the PZT-ZnO interface, the interfacial coupling produces significant enhancement on the PCE; in contrast, it causes a large decrease of the PCE.

It is helpful to analysis the ferroelectric switching process in the ITO/PZT/ZnO/Au heterostructure for better understanding the polarization-dependent interfacial coupling effect. When the semiconductor layer (e.g. ZnO) with spontaneous polarization is inserted into ITO/PZT/Au, the band structure of the system changes and thus the ferroelectric switching behavior is different from the typical electrode/ferroelectric/electrode film. On one hand, when the heterostructure is under P+ state, the accumulation of ZnO’s negative majority carriers validly screen the positive polarization charges of PZT layer at the interface, acting as the same role of an electrode. Moreover, W_D_ becomes thinner. Therefore, the switching from P+ to P− becomes easier since more parts of the applied potential can drop across the PZT layer. Consequently, the positive coercive electric field of ITO/PZT/ZnO/Au becomes smaller than that of ITO/PZT/Au (see Fig. 2(a)). On the other hand, when the heterostructure is under P- state, the situation is exactly opposite. W_D_ becomes wider and more potentials drop across the depletion layer since the resistivity of the depletion layer is nearly the same as that of PZT^15^. Thereby, the switching from P+ to P− becomes harder and needs a larger applied electric field, which agrees well with the experimental results that the negative coercive electric field of ITO/PZT/ZnO/Au becomes larger than that of ITO/PZT/Au (see Fig. 2(a)).

The polarization-dependent interfacial coupling effect can also occur when the ZnO layer is inserted between ITO and PZT (i.e. ITO/ZnO/PZT/Au). Nevertheless, its influence on the FePVE is a little more complex. From the experimental results shown in Fig. 4(d), the existence of the polarization-dependent interfacial coupling effect in ITO/ZnO/PZT/Au can be confirmed according to the evident asymmetry of photovoltaic J_sc_-V_p_ and V_oc_-V_p_ hysteresis loops. However, since the distribution of the internal electric fields in ITO/ZnO/PZT/Au is not aligned but rather E_dp-PZT_ and E_in-ZnO_ being opposite to E_bi-2_ and E_bi-3_ (Figs 2(f) and 5(c,d)), the polarization-dependent interfacial coupling effect is seriously suppressed, which causes the decrease of the modulation ratio of PCE, as shown in Fig. 3(c). In spite of this negative factor, the ITO/ZnO/PZT/Au heterostruture still exhibits great enhancement of PCE (Fig. 2(c)), which is almost the same as that of ITO/PZT/ZnO/Au. From the energy band diagrams shown in Fig. 5(c,d), the ZnO layer is irradiated in the first place by incident lights, while little light can penetrate the ZnO layer into PZT since the bandgap of PZT (E_g,PZT_ ~ 3.60 eV) is larger than that of ZnO (E_g,ZnO_ ~ 3.23 eV) (see Section III, SI). We suggest that such great enhancement of PCE in ITO/ZnO/PZT/Au can be attributed to the domination of more photo-induced carriers generated in the ZnO layer^33^.

**Figure 6:**
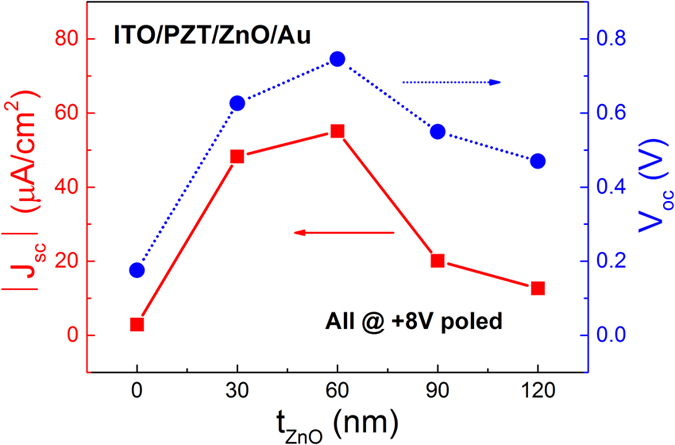
Photovoltaic J_sc_ and V_oc_ values as a function of ZnO’s thickness (t_ZnO_) in the ITO/PZT/ZnO/Au heterostructure. Note that all samples are at +8 V polarization states.

We finally studied the influence of ZnO’s thickness on the polarization-dependent interfacial coupling effect and FePVE of the PZT-ZnO heterostructures. Figure 6 presents photovoltaic J_sc_ and V_oc_ values of the ITO/PZT/ZnO/Au heterostructure measured with various ZnO thicknesses (t_ZnO_). An optimal ZnO thickness of ~60 nm is obtained, at which the heterostructure exhibits the strongest FePVE. According to our previous study^20^, the interfacial coupling strength depends on the value of spontaneous polarization (P_s_) in ZnO layer if PZT layer keeps the same thickness. On one hand, when increasing the ZnO thickness, P_s_ also increases and tends to saturate if t_ZnO_ is large enough. On the other hand, when t_ZnO_ is reduced to a critical value, P_s_ starts to drop due to the size effect. In detail, the screening charges accumulate at the surface, compensating the polarization charges to keep the discontinuity of polarization at the interface^34^. Since E_in-ZnO_ is equal to P_s_ divided by t_ZnO_, E_in-ZnO_ becomes weaker when t_ZnO_ is smaller than the critical thickness. This well explains that there exists an optimal thickness of ZnO layer where both polarization-dependent interfacial coupling effect and FePVE of the PZT-ZnO heterostructure is strongest.

## Conclusion

In conclusion, we have successfully fabricated the PZT-ZnO heterostructures consisting of ferroelectric PZT layer with reversible ferroelectric polarization and semiconductor ZnO layer with irreversible spontaneous polarization. The contact between PZT and ZnO brings about tunable polarization-dependent interfacial coupling effect, which causes significant enhancement of FePVE. The polarization-dependent interfacial coupling between PZT and ZnO layers can greatly influence the band structures of the heterostructure including the depletion layer width and the potential barrier height, which are responsible for considerable modulation of FePVE in the heterostructure. Besides, there exists an optimal ZnO layer thickness where both the polarization-dependent interfacial coupling effect and FePVE of the heterostructure are strongest. Our work provides a guide for designing ferroelectric-based solar cells and gives instructive ideas of utilizing the polarization-dependent interfacial coupling effect for developing novel optoelectronic memory devices.

## Methods

### Preparation

All the samples were prepared on commercial ITO-coated glass substrates by a sol-gel process and spin-coating technique. At first, the PZT precursor solution was obtained by using lead acetate [Pb(CH_3_COO)_2_·3H_2_O], zirconium nitrate [Zr(NO_3_)_4_·5H_2_O] and tetrabutyl titanate [Ti(C_4_H_9_O)_4_] as starting materials. Acetic acid and 2-methoxyethanol (1:4, volume ratio) were chosen as mixture solvent and a few drops of acetylacetone were added to prevent precipitation. The molar ratio of Zr/Ti was controlled to be 52/48, and 10 mol% excess lead acetate was added to compensate the loss of Pb during the annealing process. The precursor ZnO solution was obtained by mixing zinc acetate [Zn(CH_3_COO)_2_·2H_2_O] into 2-methoxyethanol. The ethanol amine was then added as stabilizer. Both two precursor solutions used in this work were 0.2 M. After being ultrasonic cleaned sequentially in deionized water, acetone and ethanol, the ITO substrate was dried by N_2_. Then the precursor PZT solution was spin-coated on the ITO substrates at 800 rpm for 8 s and 3000 rpm for 30 s in a glove box full of N_2_. Before coating the next layer, the film was dried at 280 °C for 5 min and annealed at 650 °C for 5 min under oxygen atmosphere. After that, the precursor ZnO solution was spin-coated on the PZT film in the same way except that the drying temperature was 200 °C and the annealing time was 10 min. Finally, the ITO/PZT/ZnO heterostructural film was obtained. The thicknesses of PZT and ZnO layers were controlled by repeating times of spin-coating process. For comparison purpose, ITO/ZnO/PZT and ITO/PZT structures were also fabricated in the similar above procedure.

### Characterization

The crystallographic structures of the samples were characterized by x-ray diffraction (XRD) analysis on a D/MAX-RA diffractometer with Cu Κα radiation. The cross-sectional and surface morphologies were examined by a scanning electron microscopy (SEM, ULTRA 55-44-08). Piezoresponse force microscopy (PFM, NT-MDT Inc.) images were obtained using a conductive Cr/Pt coated Si cantilever (ElectriMulti75-G, Budget Sensors) with a nominal ~3 N/m force constant and a free air resonance frequency of ~75 kHz. The transmission spectra of the samples were carried out using a UV-Vis spectrometer with a DH-2000 deuterium and halogen light source (Ommi-λ150, Zolix Inc.).

### Device Fabrication and Measurement

For electrical measurements, 40 nm Au electrodes were deposited onto the samples using ion-sputtering process through a shadow mask with the diameter of 0.2 mm. The polarization-voltage hysteresis loops were measured by a standard ferroelectric analyzer (Precision Multiferroic, Radiant Inc.). The tests were under bipolar staircase pulse of 1 kHz and the maximum external voltage reached 10 V. Photovoltaic current density vs output voltage (J-V_out_) characteristics, including dark leakage-current, open-circuit voltage and short-circuit current, were recorded by a Keithley 6517B electrometer under illumination of a 300 W ultraviolet-enhanced Xe lamp from the bottom ITO electrode, which was equipped with a UV reflector plate. The range of output spectrum is 200-400 nm (see Figure S1, Supporting Information (SI)). The light intensity was calibrated by ultraviolet light power meter. For the convenience of discussion, we define the positive voltage applied to the bottom electrode as a referential positive voltage. Similarly, the photocurrent recorded flowing through the sample from the bottom electrode to the top one was defined as positive.

## Additional Information

**How to cite this article**: Pan, D.-F. *et al*. Polarization-dependent interfacial coupling modulation of ferroelectric photovoltaic effect in PZT-ZnO heterostructures. *Sci. Rep.* **6**, 22948; doi: 10.1038/srep22948 (2016).

## Supplementary Material

Supplementary Information
