# Supplementary material for: Polarization-dependent interfacial coupling modulation of ferroelectric photovoltaic effect in PZT-ZnO heterostructures
Source: Sci Rep. 2016 Mar 8;6:22948. doi: 10.1038/srep22948 (PMC4782167; doi:10.1038/srep22948)
Supplement: Supplementary Information [file srep22948-s1.pdf]

Supplementary Information

Polarization-dependent interfacial coupling modulation of ferroelectric photovoltaic effect in PZT-ZnO heterostructures

Dan-Feng Pan[1], Gui-Feng Bi[1], Guang-Yi Chen[1], Hao Zhang[3], Jun-Ming Liu[1, 2], Guang-Hou Wang[1, 2] & Jian-Guo Wan[1, 2]

[1]National Laboratory of Solid State Microstructures and Department of Physics, Nanjing University, Nanjing 210093, China

[2]Collaborative Innovation Center of Advanced Microstructures, Nanjing University, Nanjing 210093, China

[3]Department of Physics and Astronomy, University of Kentucky, Lexington, Kentucky 40506-0055, USA

Correspondence and requests for materials should be addressed to J.G.W. (wanjg@nju.edu.cn)

I. The spectra of the incident light

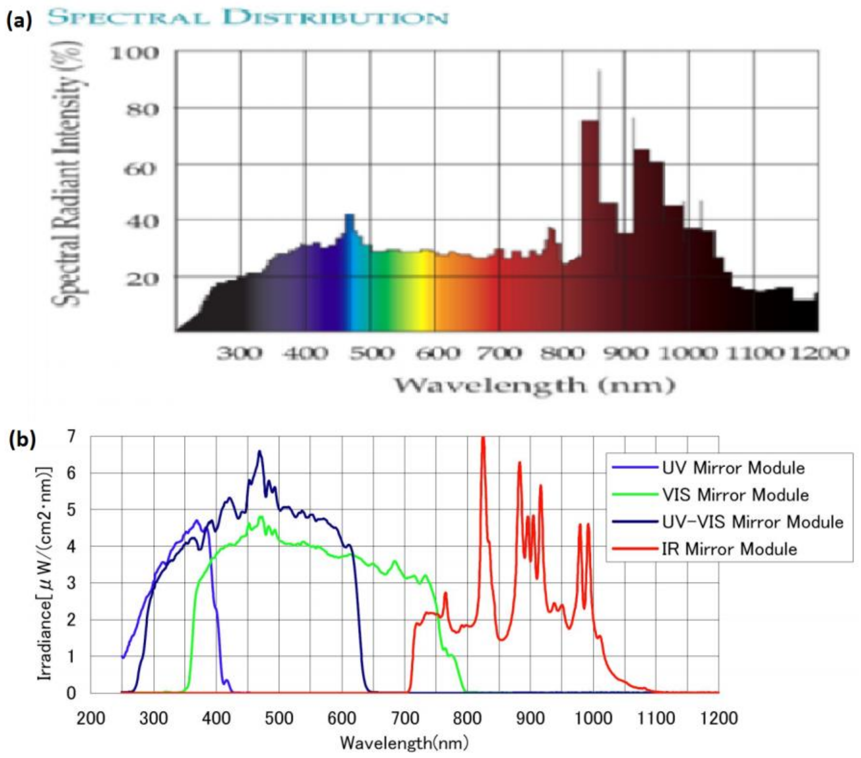

Figure S1. (a) Full spectrum distribution of our 300W ultraviolet-enhanced Xe lamp. (b) Different spectrum configurations of the output lights after equipping with all kinds of reflector plates. In this work, an UV Mirror Module was used.

II. Estimating the depolarization field and internal field directions of the as-grown PZT and ZnO layers

We separately prepared PZT and ZnO films on the ITO substrates. 40 nm Au electrodes were deposited on the films for electrical measurements. To test the polarization response to external electric fields for both films, we performed the Piezoresponse Force Microscopy (PFM) amplitude measurements. An area of 6 x 6 μm^2 was firstly written with +8V external voltage using a biased conductive tip; then a central smaller square of 3 x 3 μm^2 was reversely poled with -8V external voltage in the same way; finally the out-of-plane amplitude PFM images of 10 x 10 μm^2 area were recorded under zero bias external voltage, as depicted in the

insets of **Figures S2(a)** and **S2(b)**. Clearly, the ITO/PZT/Au exhibits switchable ferroelectric polarization response to external electric field, while the ITO/ZnO/Au only owns piezoelectric signals which can't be altered by bipolar electric field, indicating irreversible spontaneous polarization in the ZnO layer.[1]

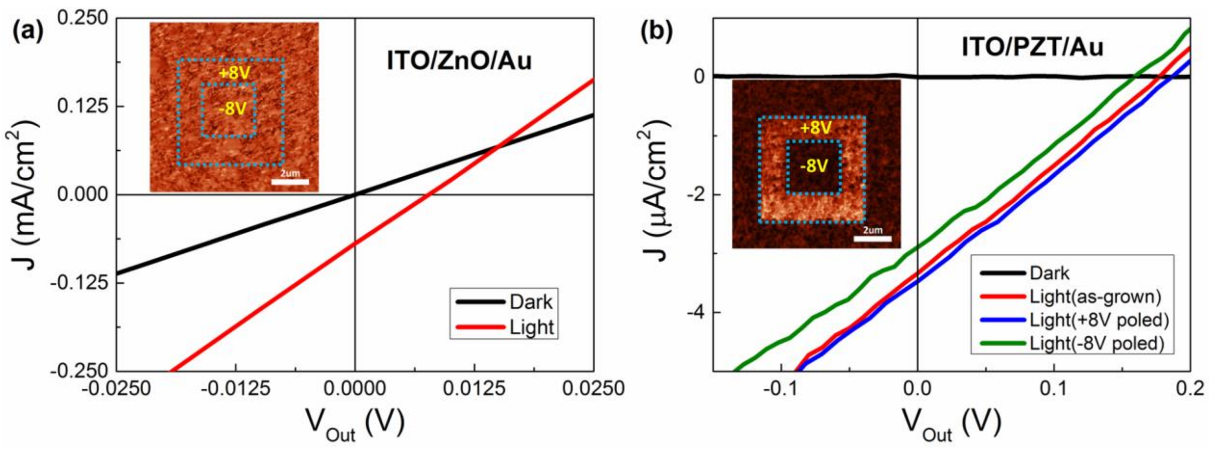

Figure S2. (a) Photovoltaic J-V_{out} curves of ITO/ZnO/Au measured under dark and light circumstances. (b) Photovoltaic J-V_{out} curves of ITO/PZT/Au measured under dark and light at different polarization states. The insets in (a) and (b) show the out-of-plane PFM images of as-grown ZnO and PZT layers, respectively.

Figure S2 presents photovoltaic current density vs output voltage (J-V_{out}) curves of the ZnO and PZT films. A very much weak photovoltaic signal is observed in the as-grown ITO/ZnO/Au sample. Note that the V_{oc} of ITO/ZnO/Au is very small, which can be ascribed to the (quasi) ohmic contacts of both ITO/ZnO and ZnO/Au interfaces due to the energy band matching between ZnO and two electrodes (see **Section IV**). When the ITO/ZnO/Au sample is short-circuited, the observed photocurrent is mainly originated from the weak internal electric field induced by the spontaneous polarization of ZnO layer. In the main body, we define the positive voltage applied to the bottom electrode as a referential positive one and so the same as the current flowing from bottom to top electrode. Then the negative J_{sc} means that

the observed photocurrent is directed to the bottom electrode, so the internal field direction of ZnO layer points to the ITO electrode. Besides, the relatively large J_{sc} value is responsible for the enhanced FePVE in the ITO/ZnO/PZT/Au heterostructure.

The situation of PZT film is similar but may be a little more complex, as seen in **Figure S2(b)**. Zheng et al. proposed a simple model to separate the Schottky barrier and polarization effects on the photocurrent of ferroelectric thin films.[2] In detail, the net internal electric fields in ferroelectric thin films comes from two parts when two electrodes are short-circuited, i.e. the depolarization field (E_{dp}) and the built-in electric fields caused by back-to-back Schottky barriers at two interfaces (E_{bi-1} and E_{bi-2}) (see **Figure 2(d)**). The negative J_{sc} value in J-V curves means that the observed photocurrent is directed to the bottom electrode, which shares the same direction with the net internal electric field. Based on the energy level diagram in **Figure 2(d)**, the net electric field can be written as:[3]

$$E_{net}=(E_{bi-1}-E_{bi-2})+|E_{dp}| \quad \text{(S1)}$$

In electrode-ferroelectric-electrode structures (e.g. ITO/PZT/Au), the depolarization field of PZT directly gives more contribution to the photocurrent while the variation of interfacial Schottky barriers have an indirect and subordinate influence on the photocurrent.[3] It means that the ferroelectric polarization effect has little influence on two interfacial Schottky barrier heights, which is totally different from the leaky $BiFeO_3$.[4] So E_{bi-1} and E_{bi-2} can be seen as the same values, no matter at what polarization direction the as-grown PZT is. It ensures the always negative J_{sc} in **Figure S2(b)**. Obviously, when the films is poled at +8V, the E_{net} is equal to $(E_{bi-1}-E_{bi-2})+E_{dp}$; while the film is poled at -8V, E_{net} is $(E_{bi-1}-E_{bi-2})-E_{dp}$. We can see that the photovoltaic J-V_{out} curve at as-grown state is more close to that of the +8V poled state, so the direction of the polarization in PZT layer at as-grown state points to the top Au electrode and the depolarization field direction in PZT layer can be identified as pointing to the bottom ITO electrode.

III. Determination of the optical band gaps of PZT and ZnO layers

Figure S3(a) gives the ultraviolet-visible transmission spectra of ITO-coated glass substrate, PZT film and PZT/ZnO bilayer film deposited on the ITO substrate, respectively. The ITO substrate has nearly 90% transmittance, so most of incident lights above 300 nm can shine directly into the deposited films.[5] To determine the optical band gaps for all the films, we carried out the fitting procedure according to the Tauc's relation:[6]

$$\alpha h\nu = A(h\nu - E_g)^m \qquad \text{(S2)}$$

where α is the absorption coefficient, hν is the photon energy, A is a constant, and m is equal to 1/2 or 2 for the direct and indirect materials, respectively. In particular, we plot the $(\alpha h\nu)^2$ versus hν curves of the three samples, and derived the optical band gaps by extrapolating the linear part of the curves to the photo energy axis at α=0, as shown in **Figure S3(b)**. Notice that the $(\alpha h\nu)^2$- hν curves near the absorption band regions are all linear, indicating the direct optical transition for the ITO, PZT and ZnO.[7] Accordingly, we obtain the optical band gaps of the ITO, PZT and ZnO to be 4.02, 3.60 and 3.23 eV, respectively.

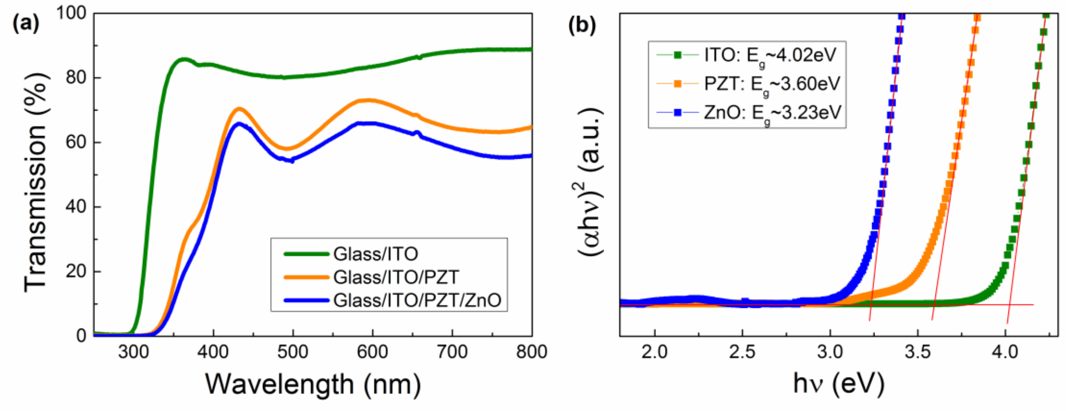

Figure S3. The optical characteristic and band gap measurements. (a) The transmission spectrums of Glass/ITO substrate, PZT and PZT/ZnO films on the ITO coated glass substrate, respectively. (b) Determination of the optical band gaps of ITO, PZT and ZnO by plots of $(\alpha h\nu)^2$ versus the photon energy hν.

IV. Determination of the barrier heights and contact formation at different interfaces

It is well known that the dark leakage current-voltage (I-V) characteristics of ferroelectric films are correlated with both bottom and top Schottky barrier heights formed at the ferroelectrics/electrode interfaces,[8] and the Schottky equation for the current density in the case of thermal emission can be expressed as follows:[9]

$$J=AT^2\exp[(\alpha V^{1/2}-q\Phi_B)/kT] \qquad \text{(S3)}$$

where J is the dark current density, A is the effective Richardson constant, T is the environmental temperature, k is Boltzmann's constant, α is positive independent of V and T, V is the applied voltage, q is the charge of an electron and Φ_B is the potential barrier height. Therefore, the top and bottom Schottky barriers can be deduced from the slopes of log (J/T^2)-1/T plots. Because of the previous definition that the positive voltages were applied to the bottom electrode, the positive branch of the leakage current vs voltage (I-V) curves is the positive V. Thus the top barrier height is determined by the positive branch and vice versa. In addition, the direction of the top build-in electric field ($E_{bi\text{-}top}$) is along with the positive voltages, which is certainly reverse to the bottom build-in electric field ($E_{bi\text{-}bottom}$).

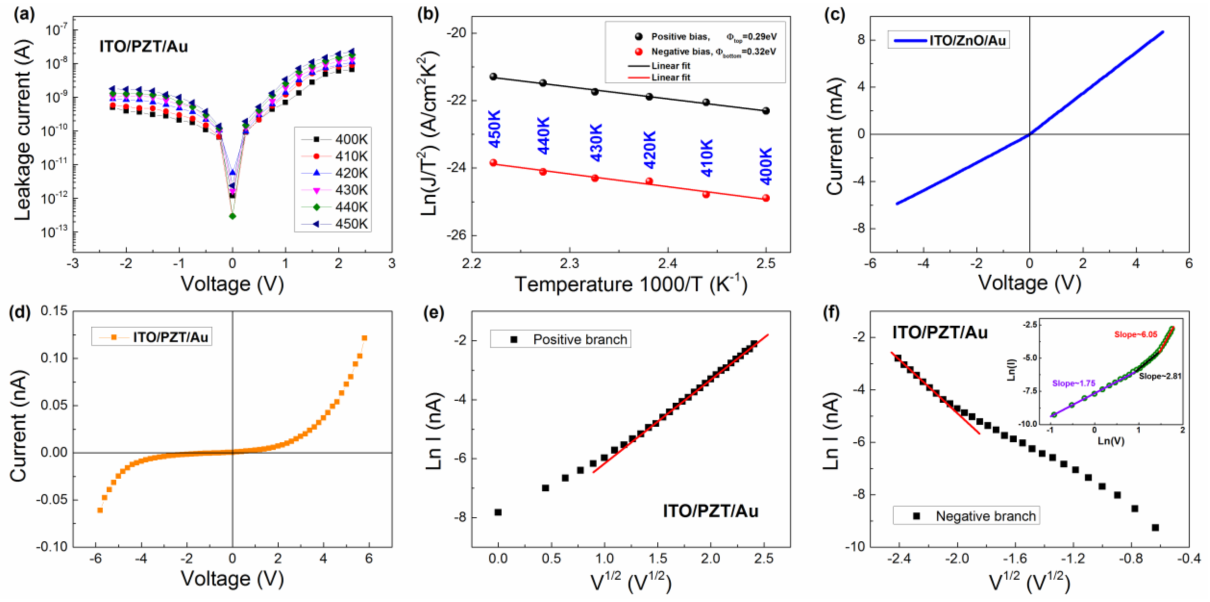

Figure S4. Experimental determinations of two Schottky barrier heights and the contact

formation of all different interfaces. (a) The dark I-V characteristics for ITO/PZT/Au sample at various 400-450K temperatures. (b) Plots of log (J/T^2)-1/T and linear fitting results for ITO/PZT/Au. (c) and (d) show the ambient temperature dark I-V measurements of the ITO/ZnO/Au sample and the ITO/PZT/Au sample, respectively. (e) Plots of Ln (I) vs. $V^{1/2}$ of the positive branch in ITO/PZT/Au sample. (f) Relevant negative branch of ITO/PZT/Au sample, the inset picture shows typical Ln (I) vs. Ln (V) curve.

Figure S4(a) shows the dark I-V characteristics of ITO/PZT/Au measured at various temperatures in the range of 400-450 K. At each temperature and every applied voltage the positive current density is always larger than the negative one, which is another evidence of the lower top Schottky barrier height than the bottom one apart from the ferroelectric polarization hysteresis. By plotting the $Ln(J/T^2)$-1/T curves and performing linear fitting, as shown in **Figure S4(b)**, we obtained the top and the bottom Schottky barrier heights to be 0.29 eV and 0.32 eV, respectively. We also performed dark I-V measurements at higher voltages for both ITO/ZnO/Au (**Figure S4(c)**) and ITO/PZT/Au (**Figure S4(d)**) samples. The measurements were done at room temperature. Both the positive and negative branches of ITO/ZnO/Au sample are observed to be linear but the two parts are not symmetric, indicating that both ZnO/Au and the ITO/ZnO interfaces have the formation of Ohmic contacts[5] but the contact resistance of ITO/ZnO is larger than that of ZnO/Au. In order to confirm the Schottky contact of ITO/PZT and PZT/Au interfaces, we also plotted the Ln(I) vs $V^{1/2}$ curves of both positive (**Figure S4(e)**) and negative (**Figure S4(f)**) branches for the ITO/PZT/Au sample. According to **Equation S3**, the Ln(I) vs $V^{1/2}$ curve should be a straight line if the conduction mechanism follows the Schottky emission.[10] From **Figure S4(e)**, the data of the positive branch of ITO/PZT/Au agree with the Schottky equation quite well, especially at high electric fields; while at negative branch it become different due to the high temperature processing of

the bottom interface.

We further plotted Ln(I) vs Ln(V) curve at the negative branch of ITO/PZT/Au, as shown in the inset of **Figure S4(f)**. At high electric fields, the current increases linearly with an exponential of slope~6.05, indicating the Schottky emission; while at lower electric fields, the slope is close to 2, which is consistent with the space-charge-limited-current (SCLC) conduction model.[11]

V. Determination of the energy band diagram for the PZT/ZnO heterostructure.

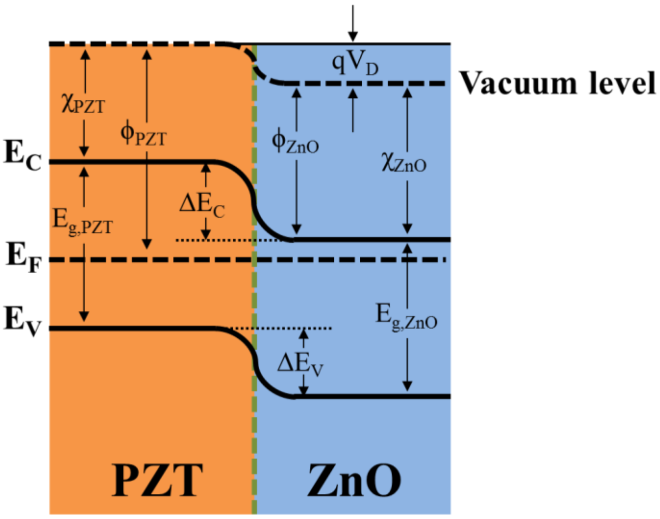

Figure S5. The simplified energy band diagram for the PZT/ZnO heterostructure. Note that the influence of polarization effect on the band structure is ignored.

According to our previous study,[12] we constructed the band structure for the PZT/ZnO bilayer as follows. To construct the band diagram for the PZT/ZnO bilayer, the electron affinities of ZnO and PZT (i.e. χ_{PZT}=3.5eV and χ_{ZnO}=4.35eV),[13] and the work function of ZnO (ϕ_{ZnO}=4.45eV)[14] were used. We suppose that the PZT Fermi level is around the middle of the energy band gap.[15] Accordingly, the work function (ϕ_{PZT}) of the PZT film is about 5.34eV. **Figure S5** shows the simplified band diagram for the PZT/ZnO bilayer. Ideally, due to the difference of the work functions between PZT and ZnO, a built-in potential V_D equal to

0.89eV is produced, which is obtained by $V_D= \phi_{PZT} - \phi_{ZnO}$. Electrons and holes are accumulated at the PZT-ZnO interface due to the existence of conduction and valence band offsets. To calculate the barrier heights for electrons and holes in such a p-n junction, the band offsets have to be taken into account. The offset of the conduction band is ΔE_C= 0.85eV according to $\Delta E_C= \chi_{ZnO} - \chi_{PZT}$. The band offset of the valence band is denoted by $\Delta E_V= \chi_{ZnO} - \chi_{PZT} + E_{g,ZnO} - E_{g,PZT}$. According to **Section III**, the $E_{g,ZnO}$ and $E_{g,PZT}$ are determined to be 3.23eV and 3.60eV. Thus ΔE_V equals to 0.48eV.

References

1. Shi J, Starr MB, Wang X. Band Structure Engineering at Heterojunction Interfaces via the Piezotronic Effect. *Advanced Materials* **24**, 4683-4691 (2012).
2. Zheng F, Xu J, Fang L, Shen M, Wu X. Separation of the Schottky barrier and polarization effects on the photocurrent of Pt sandwiched Pb(Zr0.20Ti0.80)O3 films. *Applied Physics Letters* **93**, 172101 (2008).
3. Cao D, *et al.* Polarization effect on the photocurrent of Pt sandwiched multi-crystalline ferroelectric films. *Materials Chemistry and Physics* **129**, 783-786 (2011).
4. Cai W. et al. Switchable diode effect and ferroelectric resistive switching in epitaxial $BiFeO_3$ thin films. *Applied Physics Letters* **98**, 192901 (2011).
5. Cao D, *et al.* High-Efficiency Ferroelectric-Film Solar Cells with an n-type Cu2O Cathode Buffer Layer. *Nano Letters* **12**, 2803-2809 (2012).
6. Tauc J. Absorption edge and internal electric fields in amorphous semiconductors. *Materials Research Bulletin* **5**, 721-729 (1970).
7. Lu H, Pan JS, Chen XF, Zhu WG, Tan OK. Influence of annealing temperature on the

band structure of sol-gel Ba0.65Sr0.35TiO3 thin films on n-type Si(100). *Applied Physics Letters* **88**, 132907 (2006).

8. Qin M, Yao K, Liang YC, Gan BK. Stability of photovoltage and trap of light-induced charges in ferroelectric WO3-doped (Pb0.97La0.03)(Zr0.52Ti0.48)O3 thin films. *Applied Physics Letters* **91**, 092904 (2007).
9. Pintilie L, Vrejoiu I, Hesse D, LeRhun G, Alexe M. Ferroelectric polarization-leakage current relation in high quality epitaxial Pb(Zr, Ti)O3 films. *Physical Review B* **75**, 104103 (2007).
10. Hu W, *et al.* Opportunity of Spinel Ferrite Materials in Nonvolatile Memory Device Applications Based on Their Resistive Switching Performances. *Journal of the American Chemical Society* **134**, 14658-14661 (2012).
11. Rose A. Space-Charge-Limited Currents in Solids. *Physical Review* **97**, 1538-1544 (1955).
12. Zhou M-X, Li Z-W, Chen B, Wan J-G, Liu J-M. Tunable resistive switching behaviour in ferroelectric–ZnO bilayer films. *Journal of Physics D: Applied Physics* **46**, 165304 (2013).
13. E. Cagin, *et al*. Hysteretic metal-ferroelectric-semiconductor capacitors based on PZT/ZnO heterostructures. *Journal of Physics D: Applied Physics* **40**, 2430 (2013).
14. Fang Y. J., *et al*. Behind the change of the photoluminescence property of metal-coated ZnO nanowire arrays. *Applied Physics Letters* **98**, 033103 (2011).
15. Yang H., *et al*. Rectifying current-voltage characteristics of $BiFeO_3$/Nb-doped $SiTiO_3$ heterojunction. *Applied Physics Letters* **92**, 102113 (2008).
